# Draft hybrid assembly and annotation of the *Drosophila palustris and Drosophila subpalustris* genomes

**DOI:** 10.1093/g3journal/jkag226

**Published:** 2026-08-19

**Authors:** William Dion, Emily Washeleski, Tessa E Steenwinkel, Emily Taylor, Prajakta P Kokate, Aidan VanDrie, Thomas Werner, Paul D Goetsch

**Affiliations:** School of Health, Human, and Biological Systems, Michigan Technological University, Houghton, MI 49931, United States; School of Health, Human, and Biological Systems, Michigan Technological University, Houghton, MI 49931, United States; School of Health, Human, and Biological Systems, Michigan Technological University, Houghton, MI 49931, United States; School of Health, Human, and Biological Systems, Michigan Technological University, Houghton, MI 49931, United States; School of Health, Human, and Biological Systems, Michigan Technological University, Houghton, MI 49931, United States; School of Health, Human, and Biological Systems, Michigan Technological University, Houghton, MI 49931, United States; School of Health, Human, and Biological Systems, Michigan Technological University, Houghton, MI 49931, United States; School of Health, Human, and Biological Systems, Michigan Technological University, Houghton, MI 49931, United States

**Keywords:** Drosophilidae, comparative genomics, hybrid assembly, synteny, phylogenomics, quinaria species group

## Abstract

Species within the quinaria group of the genus *Drosophila* are models for ecological genetic studies on topics that include morphological diversity, color pattern development, and feeding behavior. *Drosophila palustris* and *Drosophila subpalustris* are closely related members within the quinaria species group that differ primarily in subtle morphological and behavioral traits. To support future evolutionary genomic studies in drosophilids, we completed new draft hybrid assemblies and annotations of the *D. palustris* and *D. subpalustris* genomes. We obtained a fragmented assembly for *D. palustris* (N50 = 874 Kb) and a near chromosome-scale *D. subpalustris* assembly (N50 = 27.5 Mbp). Assembly contiguity, repeat content, and gene annotation compared well to other published quinaria group genomes. To demonstrate the utility of our hybrid assemblies, we performed whole-genome synteny and phylogenomic comparisons within the quinaria clade. When compared to the chromosome-level *Drosophila innubila* assembly, the median size of syntenic blocks among quinaria group genomes was approximately 1 Mbp, although this estimate was influenced by assembly contiguity. Phylogenomic analyses estimated that the 2 major branches of the quinaria group diverged ∼17.9 MYA, with *D. palustris* and *D. subpalustris* diverging ∼1 to 2 MYA. We expect that our *D. palustris* and *D. subpalustris* assemblies will provide important additions to studies of genome evolution within Drosophilidae.

## Introduction

Members of the recently evolutionarily diverged quinaria adaptive radiation within the genus *Drosophila* have served as models to study metabolism (Steenwinkel et al. 2022), feeding behavior (Scott Chialvo et al. 2019), toxin tolerance (Kokate et al. 2022; Erlenbach et al. 2023), and phylogenetics (Finet et al. 2021). Species of the quinaria group also exhibit complex body and wing color patterning (Werner et al. 2010; Koshikawa 2020). We are interested in 2 members of the quinaria group, *Drosophila palustris* and *Drosophila subpalustris* (Fig. 1). Both species are non-mycophagous, inhabit the eastern and midwestern United States, and reproduce in rotting water plants (Spicer and Jaenike 1996). However, *D. palustris* and *D. subpalustris* display some deviation in courtship behavior (Grossfield 1972) and color patterning (Dion et al. 2020).

The combination of long-read Oxford Nanopore Technologies (ONT) data with short-read Illumina reads for polishing recently delivered high-quality and cost-effective assemblies for drosophilid genomes (Kim et al. 2021; Kim et al. 2024). Kim et al. (2021) generated 101 highly contiguous *de novo* assemblies from 93 laboratory lines, using an ONT-centric hybrid workflow. Follow-up work demonstrated that the same hybrid strategy produced chromosome-scale assemblies from single wild adult flies (Kim et al. 2024). Together, these studies proved that a hybrid assembly method generates near-reference quality genomes, enabling a more affordable and scalable platform for expanding genomic resources across drosophilids.

Despite the recent advances in hybrid *de novo* genome sequencing and assembly of drosophilid genomes, no genomic resources are available for *D. palustris* and *D. subpalustris.* Out of 32 species described in the quinaria group (Bächli 2026), we identified 9 medium- to high-quality assemblies (Hill et al. 2019; Kim et al. 2024; Obbard et al. 2024). To contribute to the expanding genome resources in the clade, we present draft hybrid assemblies and annotations of the *D. palustris* and *D. subpalustris* genomes. Our genomes were assembled by combining Nanopore-based long-read sequences with Illumina-based short-read sequences from genomic DNA. Following assembly and annotation of our genomes, we compared the quality of our assemblies to the previously published genomes from the quinaria group. We then performed comparative analyses across the quinaria group to demonstrate that *D. palustris* and *D. subpalustris* diverged ∼1 to 2 MYA. Altogether, we intend that these genome assemblies will provide a valuable resource for researchers interested in understanding the evolution of color patterning, metabolism, and toxin tolerance across the quinaria adaptive radiation and genome evolution in general across Drosophilidae.

## Materials and methods

### Sampling and genomic DNA extraction

*D. palustris* was collected by Tessa Steenwinkel in Waunakee, Wisconsin. *D. subpalustris* was purchased from the *Drosophila* Species Stock Center, stock number 15130–2071.00. Fly stocks for both species were maintained on cornmeal-sucrose-yeast medium at room temperature (Werner et al. 2018).

We performed 2 high molecular weight genomic DNA extractions for each species because the first sequencing library set sequenced poorly (Table 1). The first genomic DNA prep was performed from 25 adult male flies using a phenol:choroform:isoamyl alcohol extraction and ethanol precipitation protocol. The second prep followed the DNA extraction protocol demonstrated in Kim et al. (2021) with minor adjustments. Briefly, we flash-froze 100 to 200 adult male flies in Homogenization Buffer (30 mM Tris–HCl pH 8.0, 100 mM NaCl, 10 mM EDTA, 0.5% Triton X-100) and stored them at −80 °C. Thawed flies were homogenized on ice in a 2-mL Kimble Kontes Dounce homogenizer 4-5 times with pestle A and 4-5 times with pestle B. Following centrifugation to clear large debris, nuclei were pelleted with a 2,000 × *g* centrifugation at 4 °C. The nuclei-containing pellet was resuspended in 200 µL Homogenization Buffer before adding 1,300 µL Extraction Buffer (100 mM Tris–HCl pH 8.0, 100 mM NaCl, 20 mM EDTA, 0.2% SDS) and 30 ng Proteinase K and incubated at 37 °C for 2 to 4 h. We performed one phenol:chloroform:isoamyl alcohol extraction on the nuclei lysate followed by one back extraction of the organic layer with one volume of Extraction Buffer and one back extraction of the combined aqueous later with one volume of chloroform. Finally, we performed an ethanol precipitation of the combined aqueous sample, resuspending the final pellet in 0.1× TE and storing the sample at 4 °C for sequencing library preparation. We quantified DNA concentration with the Qubit dsDNA HS Assay Kit (ThermoFisher Scientific) and Nanodrop to confirm absorption ratios of 260/280 greater than 1.8 and 260/230 greater than 2.0 for all genomic DNA samples.

### Oxford nanopore long-read sequencing of genomic DNA

Before prepping sequencing libraries from both sets of genomic DNA extractions, we purified high molecular weight genomic DNA using 0.5×—0.6× volumes of SeraPure beads (Rohland and Reich 2012). We followed standard protocols for the Oxford Nanopore Technologies (ONT) Rapid Sequencing Kit (SQK-RAD004) for the first sequencing library and standard protocols for the ONT Ligation Sequencing Kit (SDK-LSK109) for the second sequencing library. Each library was sequenced on an individual R 9.4.1 MinION flow cell.

### Illumina short-read genomic DNA and RNA sequencing

Genomic DNA libraries for Illumina short-read sequencing were processed from the second extraction described above, following standard protocols for the NEBNext Ultra II FS kit (New England Biolabs). In parallel, RNA was extracted using a standard Trizol–chloroform phase separation followed by isopropanol precipitation from 10 to 20 mixed male and female adult flies. We generated RNA-sequencing libraries from a total of 1 µg purified RNA, using the NEBNext Poly(A) mRNA Magnetic Isolation Module and NEBNext Ultra II Directional Library Prep Kit for Illumina (NEB). DNA- and RNA-seq libraries were multiplexed using the NEBNext Multiplex Oligos for Illumina (NEB). DNA- and RNA-seq libraries were sequenced on an Illumina HiSeq X system by Novogene Corporation Inc. (Davis, CA, USA) to acquire 150-bp paired-end reads.

### Genome assembly

Raw nanopore fast5 data files from both sequencing runs were base-called together, using Guppy v6.5 with the SUP model configuration file option -c dna_r9.4.1_450bps_sup.cfg. We used MinIONQC v1.4.1 (Lanfear et al. 2019) to assess Nanopore read quality for both the initial FAST model called reads and the combined SUP model called reads (Table 1). We estimated genome size using Oxford Nanopore sequencing reads. The reads were quality-filtered with filtlong v0.2.1, available at https://github.com/rrwick/Filtlong, using the parameters –min_length 1,000 –keep_percent 75 –min_mean_q 20, retaining the top 75% of reads by quality. K-mer frequencies (k = 21) were generated with jellyfish (Marçais and Kingsford 2011) count -C -m 21. Genome size and heterozygosity were estimated using GenomeScope (Vurture et al. 2017) based on the resulting k-mer distribution.

To ensure that our newly generated genomes are comparable to previously published resources, we replicated the genome assembly pipelines established by Kim et al. (2021) and Erlenbach et al. (2025). We created an initial assembly using Flye v2.9.2 (Kolmogorov et al. 2019) and the -nano-raw setting. We aligned reads to the initial draft genome with minimap v2.26 (Li 2018) and the map-ont setting, then the initial draft was polished using Racon v1.5 (Vaser et al. 2017) with settings -m 8 -x 6 -g −8 -w 500. The alignment and polishing steps were repeated once more, before the assembly was polished using medaka v1.11.1 and default parameters. Illumina short reads were aligned to the racon(x2)/medaka polished draft, using minimap v2.26 and the -ax sr setting, before polishing 2 times iteratively using Pilon v1.24 (Walker et al. 2014) with settings -fix snps,indels.

We ran BUSCO v5.5.0 (Simão et al. 2015) to assess assembly completeness. We observed that the *D. palustris* assembly had >5% complete and duplicated BUSCOs, which is why we performed haplotig identification and removal by applying purge_haplotigs v1.1.2 (Roach et al. 2018), following suggested parameters on the original Flye *D. palustris* assembly. Following haplotig removal, the *D. palustris* assembly was polished using the Racon(x2)/medaka/pilon(x3) pipeline described earlier. Contigs were checked for contamination using blastn v2.17.0 (Camacho et al. 2009), and any identified microbial sequences were removed. We also removed contigs shorter than one kilobase from the final assembly. We used QUAST v5.2.0 to assess genome assembly quality (Gurevich et al. 2013). We calculated sequencing coverage using mosdepth v0.3.3 (Pedersen and Quinlan 2018) after mapping raw Nanopore and Illumina reads to the finished assembly, using minimap v2.26 and BWA v0.7.17 (Li and Durbin 2009).

### Genome annotation of repeat regions and genes

To identify repetitive sequences, we ran RepeatMasker v4.1.5 (Tarailo-Graovac and Chen 2009) on the final assembled genome, using the *Drosophila* genus-specific repeat library (-species drosophila_flies_genus) and -xsmall flag to softmask repeats. To generate preliminary gene annotations, we ran BRAKER v3.0.6 (Stanke et al. 2006, 2008) on the softmasked genome assembly. RNA-seq reads from each species were aligned to the genome assembly using STAR v2.7.11b (Dobin et al. 2013) with the –outSamstrandField intronMotif option enabled. We ran BRAKER on the softmasked genome assembly with the RNA-seq input, resulting in an execution using the Evidence-based training (ET) mode (Gabriel et al. 2021).

### Genome comparisons and phylogenomic analysis

We assessed genome completeness using BUSCO with the *diptera_odb10* lineage dataset to evaluate the presence of conserved single-copy orthologs. BUSCO complete hits were used to map contigs to putative chromosomes using the *D. innubila* chromosomal genome assembly as reference (Hill et al. 2019). We calculated syntenic feature lengths across placed contigs relative to the *D. innubila* reference using chromeister v1.5.a (Pérez-Wohlfeil et al. 2019). We generated a multigenome synteny riparian plot using the R package GENESPACE v.1.4 (Lovell et al. 2022) using shared single-copy BUSCO genes for *D. palustris, D. subpalustris, D. innubila,* and *D. melanogaster*. *D. palustris* and *D. subpalustris* contigs mapped to putative Muller elements were ordered and concatenated in descending size order.

Phylogenomic relationships were inferred using the BUSCO phylogenomics pipeline, available at https://github.com/jamiemcg/BUSCO_phylogenomics. The resulting concatenated amino acid supermatrix was back-translated using PAL2NAL v14.1 (Suyama et al. 2006) to generate a full codon supermatrix and a standalone third-codon position supermatrix. We prepared our phylogenetic data for Bayesian divergence time inference using the IQ-TREE v3.1.2 (Wong et al. 2026) –dating mcmctree flag. Substitution parameters were explicitly modeled for each matrix using the JTT + F + G4 amino acid model, GY + F3X4 codon model, and GTR + F + G4 third-codon position model. Absolute divergence times and posterior confidence intervals were calculated using MCMCtree within PAML v4.10.10 (Yang 2007; dos Reis and Yang 2011) and the correlated-rates relaxed-clock option (clock = 3). Calibration points for the divergence estimates were obtained from TimeTree (Kumar et al. 2022) and summarized in Table 5. The generated phylogenic trees were visualized using FigTree v1.4.4.

## Results and discussion

### Genome assembly metrics

We sequenced high molecular weight genomic DNA using both Oxford Nanopore Technologies (ONT) rapid and ligation kits on separate genomic DNA purification preparations from pools of *Drosophila palustris* and *Drosophila subpalustris* males. The ligation kit yielded ∼6-fold more raw data compared to the rapid kit, which we attributed to the quality of genomic DNA purification. We combined both sequencing runs together for each species and re-ran base-calling with the super accurate (SUP) base-calling model, yielding 13.8 Gbp of long read data for *D. palustris* and 21.1 Gbp of long read data for *D. subpalustris*. The N50 read length was 8.9 kb for *D. palustris* and 6.3 kb for *D. subpalustris*. In parallel, we sequenced 7.1 Gbp and 21.3 Gbp of Illumina short paired-end reads for each respective species (Table 1). Based on k-mer analysis of filtered higher-quality Nanopore reads, we estimated genome sizes were approximately 174 Mbp for *D. palustris* and 179 Mbp for *D. subpalustris.*

**Table 1.** Summary statistics of genomic DNA sequencing data.

D. palustris	ONT Run 1	ONT Run 2	ONT SUP	ONT Q ≥ 7	Illumina
# reads	1,098,754	2,224,182	3,322,927	3,055,663	23,623,074
Raw data (Gbp)	1.87	12.41	14.73	13.8	7.1
N50 Length (bp)	4,972	9,291	8,703	88,902	-
Max length (bp)	106,676	155,431	157,823	157,823	-
Median Q value	9.2	10.6	14	14.3	-
*D. subpalustris*					
# reads	3,498,810	5,307,665	8,806,475	8,167,330	71,166,246
Raw data (Gbp)	3.36	17.96	22.3	21.1	21.3
N50 Length (bp)	1,653	8,180	6,126	6,279	-
Max length (bp)	56,778	433,225	395,019	282,152	-
Median Q value	8.2	10.5	13.2	13.5	

Metric are detailed across 2 individual Oxford Nanopore Technologies (ONT) long-read sequencing runs, combined cumulative ONT data basecalled with the super-accuracy basecalling (SUP) model, a subset of reads used for the assembly with a quality score threshold of Q ≥ 7, and paired-end Illumina short-read sequencing data.

Abbreviations: Gbp, gigabase pairs; bp, base pairs; N50, sequence length threshold where 50% of the total read volume is contained within reads of equal or greater length; Median Q, median Phred-scaled base quality score.

We generated contig-level assemblies from the long-read ONT sequences using Flye and then used short-read Illumina sequences for polishing. The final *D. palustris* assembly contained 1,151 contigs that were approximately 186.4 Mbp in length with an N50 of 874 kbp. The final *D. subpalustris* assembly contained 1,553 contigs that were approximately 172.6 Mbp in length with an N50 of 27.5 Mbp. Our assemblies resulted in a high benchmarking universal single-copy ortholog (BUSCO) genome completeness score of 99.1% with a 2.9% duplication rate for *D. palustris* and 97.1% with a 0.4% duplication rate for *D. subpalustris*. Mapping raw reads to the final assembly indicated that our sequencing coverage was approximately 11× (Illumina) and 64.3× (Nanopore) for a total of 75.3× for *D. palustris* and 34.6× (Illumina) and 102.2× (Nanopore) for a total of 136.8× for *D. subpalustris* (Table 2).

**Table 2.** Summary statistics for draft genome assemblies.

	*D. palustris*	*D. subpalustris*
Assembly length (bp)	186,382,118	172,555,552
Contig #	1,151	1,553
Largest contig (bp)	7,786,370	33,305,132
Contig N50 (bp)	874,281	27,511,894
Contig L50	51	3
Average GC content (%)	37.11	37.02
BUSCO (genome)	C:99.1%[S:96.2%,D:2.9%] F:0.4%,M:0.5%,n:3285	C:97.1%[S:96.7%,D:0.4%] F:0.3%,M:2.6%,n:3285
Total sequencing coverage	75.32x	126.84x
Heterozygosity (%)	1.77	0.432

Final assembly profiles generated using a hybrid pipeline combining Oxford Nanopore long reads and Illumina short reads. Genomic completeness was assessed using Benchmarking University Single-Copy Orthologs (BUSCO) evaluated against the *diptera_odb10* lineage dataset (*n* = 3,285 orthologs).

Abbreviations: bp, base pairs; N50, contig length threshold at which 50% of the total genome length is covered by contigs of equal or greater size; L50, the minimum number of contigs required to constitute 50% of the total genome length; GC, guanine-cytosine nucleotide content ratio; C, complete orthologs; S, single-copy orthologs; D, duplicated orthologs; F, fragmented orthologs; M, missing orthologs; Heterozygosity (%), percentage of sequence divergence between homologous chromosomes estimated via k-mer profiling using GenomeScope.

Our final assemblies for *Drosophila palustris* and *Drosophila subpalustris* compared well with previously published genomes in the *Drosophila* quinaria species group and the model *Drosophila melanogaster* genome. We next compared our genomes to published genomes from additional quinaria group members: *D. falleni, D. kuntzei, D. phalterata, D. quinaria, D. recens, D. rellima, D. suboccidentalis, D. subquinaria,* and *D. innubila* (Hill et al. 2019; Kim et al. 2024; Obbard et al. 2024). To ensure a direct comparison, we split chromosome- and scaffold-level assemblies to the contig level. Our data demonstrate that hybrid assembly for *D. subpalustris* achieved a near-complete chromosomal assembly on par with the *D. melanogaster* contig-level data, with 5 contigs representing most of the assembly (Fig. 1b and c).

**Fig. 1.**
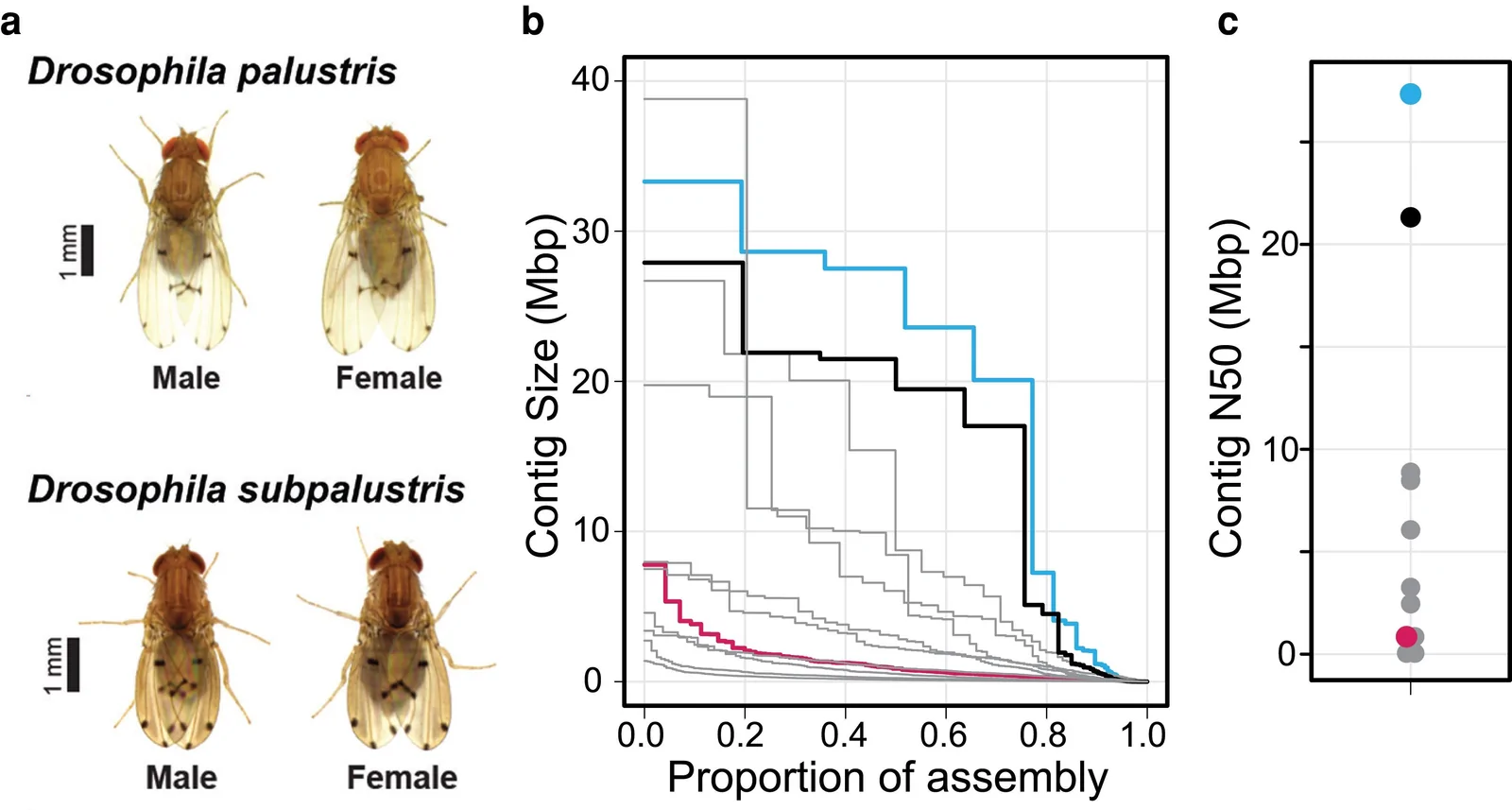
a) Representative images of male and female *Drosophila palustris* and *Drosophila subpalustris,* 1 mm scale bar. Images reproduced from (Werner et al. 2018), licensed under a CC BY-NC-ND 4.0 license. b and c) Contig level contiguity of *Drosophila palustris* (magenta) and *Drosphila subpalustris* (cyan) was compared to *Drosophila melanogaster* (black) and other members of the quinaria species group (grey). b) The Nx curve displays the size of each contig in Mbp (y-axis) sorted in descending order in relation to the cumulative proportion of the genome assembly covered (x-axis) for each species. c) Stripchart of the size of the contig at which 50% of the assembly is covered (Contig N50) for each species.

However, our *D. palustris* hybrid assembly showed lower continuity compared to similarly sequenced genomes, which we attributed to the lower sequencing coverage combined with high genome-wide heterozygosity (Fig. 1b and c). Using GenomeScope, we observed a high heterozygosity rate of 1.77% for *D. palustris,* which contrasts with the low heterozygosity rate of 0.432% observed for *D. subpalustris* (Table 2). Genomic heterozygosity rates exceeding 1% are a well-documented barrier for generating highly contiguous assemblies (Kajitani et al. 2014). The observed heterozygosity differences between species reflect the history of the laboratory strains. Our *D. subpalustris* stock was sub-cultured for many years before material for sequencing was collected, allowing for extensive purging of genetic variation. In contrast, our *D. palustris* samples were prepared for sequencing less than a year after the strain was isolated from the field, preserving its natural wild heterozygosity and thus impacting the overall assembly continuity. Regardless, our *D. palustris* genome assembly meets or exceeds the continuity of at least 4 other previously published quinaria group genomes (Fig. 1b and c).

### Genome repeat analysis and draft gene annotation

Repeat analysis using the RepeatMasker software softmasked ∼20.0 Mbp (10.76%) of the *D. palustris* genome and ∼16.8 Mbp (9.76%) of the *D. subpalustris* genome as interspersed repeats and low-complexity DNA sequence. Across both species, we estimated total repeat content of 14.12% in *D. palustris* and 12.35% in *D subpalustris*. Repeats in both genomes consist primarily of retroelements (LINEs and LTR elements), DNA transposons, rolling-circle elements, interspersed repeats, small RNAs, simple repeats, and low complexity regions. A complete breakdown of values for each repeat class is provided in Table 3. The values are low compared to other *Drosophila* species (Sessegolo et al. 2016) but are similar to the 13.5% total reported for *D. innubila* (Hill et al. 2019). Altogether, our results suggest that genomes of quinaria group species contain a reduced level of repeat content.

**Table 3.** Repeat analysis.

Annotation	*D. palustris (%)*	*D. subpalustris (%)*
LINEs	0.87	0.86
LTR Elements	2.25	1.50
DNA transposons	0.24	0.23
Rolling-circles	0.64	0.68
Interspersed repeats	3.37	2.60
Small RNA	0.01	0.02
Simple repeats	5.76	5.51
Low complexity	0.98	0.95
Total	14.12	12.35

Percentages represent the proportion of the final assembled genome length occupied by each respective repeat region class.

Abbreviations: LINEs, Long Interspersed Nuclear Elements; LTR, Long Terminal Repeat retrotransposons; DNA transposons, Class II DNA transposable elements; Rolling circle, Helitron-like rolling-circle transposons; Small RNA, structural non-coding small RNA elements; Simple repeats, short microsatellite tandem arrays; Low complexity, regions of highly biased or single-nucleotide composition; Total, the cumulative percentage of the assembly classified as repeat content.

We annotated protein-coding genes in *D. palustris* and *D. subpalustris* using repeat-masked assemblies and evidence-guided prediction using BRAKER (Stanke et al. 2006, 2008; Gabriel et al. 2021). For evidence, we incorporated RNA-seq data from mixed male and female adult flies along with conserved proteins in the *Drosophila* genus. Total RNA-seq input exceeded 56 million reads per replicate for *D. palustris* and 42 million reads per replicate for *D. subpalustris* (Table 4). We identified 20,687 predicted protein-coding genes in *D. palustris* and 17,716 genes in *D. subpalustris*. Mean gene lengths were 3,449 bp and 3,831 bp, respectively. Our annotations demonstrate high alignment with our RNA-seq evidence, as 94.25 and 92.91% of reads uniquely mapped across 2 replicates for *D. palustris* and 86.64 and 89.67% of reads uniquely mapped across 2 replicates for *D. subpalustris*. BUSCO analysis of the annotated protein-coding genes also indicated a high completeness for both annotations, with *D. palustris* at 97.2% and *D. subpalustris* at 95.8% completeness. We observed similar gene architecture in our draft annotations between the 2 species, and additional metrics including CDS length, exon and intron lengths, and exons per CDS are summarized in Table 4. In comparison, *D. innubila* mean gene length was 4,350 bp (Hill et al. 2019). Overall, gene annotations in the quinaria species group are limited, so our draft analysis provides an initial benchmark for the clade.

**Table 4.** Summary statistics for gene annotation.

	*D. palustris*	*D. subpalustris*
Total RNA-seq reads (Rep 1, Rep 2)	64,325,877, 56,363,749	42,617,992, 43,677,947
Uniquely Mapped Reads % (Rep 1, Rep 2)	94.25, 92.91	86.64, 89.67
Predicted genes	20,687	17,716
Mean gene length (bp)	3,449	3,831
Mean CDS length (bp)	1,400	1,512
Average exons per CDS	3.5	3.7
Average exon length (bp)	378	371
Average intron length (bp)	836	909
BUSCO (genes)	C:97.2%[S:77.9%,D:19.3%] F:1.1%,M:1.7%,n:3285	C:95.8%[S:77.6%,D:18.2%] F:0.7%,M:3.5%,n:3285

Gene annotation statistics including total input transcriptomic data, mapping efficiencies, predicted protein-coding genes, mean sequence lengths, and exon/intron lengths for *Drosophila palustris* and *Drosophila subpalustris.* Total RNA-seq reads and final mapping rates are reported individually for both biological replicates (Rep 1, Rep 2). Annotation completeness was verified using Benchmarking Universal Single-Copy Orthologs (BUSCO) evaluated at the transcript level against the *diptera_odb10* lineage database (*n* = 3,285).

Abbreviations: Rep, replicate, bp, base pairs; CDS, coding sequence; C, complete orthologs; S, single-copy orthologs; D, duplicated orthologs; F, fragmented orthologs; M, missing orthologs.

### Contig assignment to putative chromosome and global synteny analysis

*Drosophila* genomes generally have 4 chromosomes, often described as 6 Muller elements A-F representing the chromosomes X, 2L, 2R, 3L, 3R, and 4, respectively (Muller and Huxley 1940). Although gene position within each Muller element may differ between *Drosophila* species, genes rarely transfer between Muller elements (Ranz et al. 2001; Kim et al. 2021). We took advantage of the *D. innubila* chromosomal assembly and the high BUSCO completeness within the 10 other sequenced quinaria group species (Fig. 2a) to assign contigs to a putative chromosome (Supplementary Table 1). We compared the chromosomal locations of BUSCO genes in the *D. innubila* reference to their contig locations in each other genome. Contigs were assigned to a Muller element based on the majority consensus of their mapped BUSCO genes. Among all analyzed assemblies, 59.8–92.3% of total assembly length was assigned to a Muller element, with variation largely reflecting differences in contiguity of the assemblies (Fig. 2b).

**Fig. 2.**
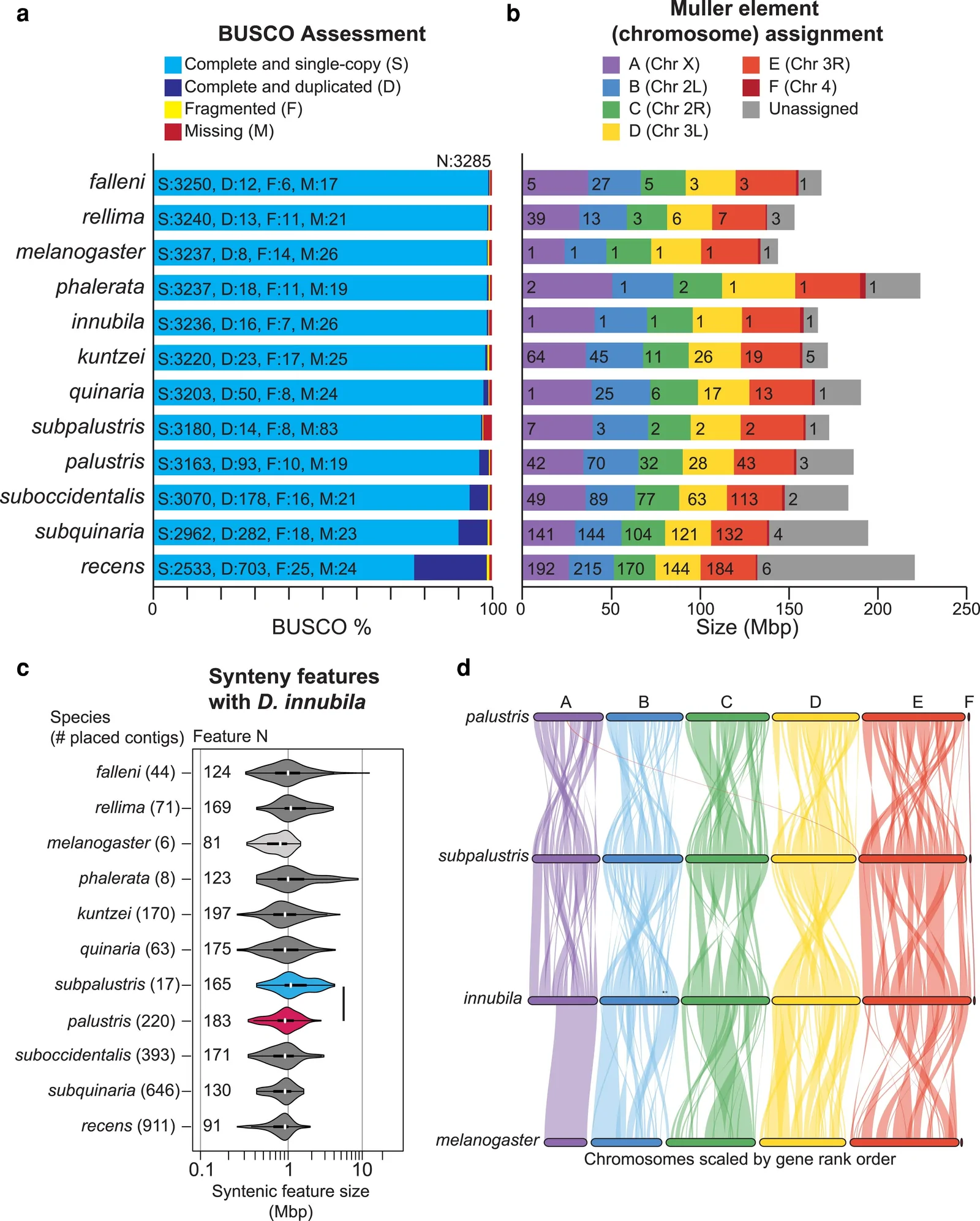
a) Completeness of the assembly as determined by BUSCO v5.5.0 of selected quinaria group species. Individual assembly summary statistics provided. The x-axis displays the percentage of total BUSCO IDs (diptera_odb10, *N* = 3285) that are complete and single copy (S), complete and duplicated (D), fragmented (F), and missing (M) in each assembled genome. b) Bar chart of assembly contig assignments to putative chromosomes. Contigs are plotted by size (Mbp) on the x-axis and stacked by Muller element. The number of assigned contigs in each assembly for each Muller element is presented on the left side of each bar segment, except for Muller element F (chromosome 4), which is presented to the right. c) Violin plot of synteny feature size in Mbp between each indicated *Drosophila* species and *D. innubila.* The data are plotted on a log10 scale. Violin shapes indicate the full distribution density, and the white bar indicates the median. Distributions for *D. palustris* and *D. subpalustris* are highlighted in magenta and cyan, respectively. Statistical significance between their distributions was calculated using a Mann–Whitney U test (** *P*-value < 1 × 10^−5^). d) GENESPACE riparian plot mapping syntenic connections within Muller elements based on shared BUSCO single-copy orthologs among *D. palustris*, *D. subpalustris*, *D. innubila*, and *D. melanogaster*. Ribbons connect orthologous genes, with chromosomes scaled by gene rank order and color-coded by Muller element assignment. *D. innubila* served as the reference genome. *D. palustris* and *D. subpalustris* contigs assigned to each Muller element were arranged sequentially by size. Because these contigs are not in their chromosomal order, the plot does not display true structural variants among *D. palustris, D. subpalustris*, and *D. innubila*.

For *D. palustris,* 82.7% of the assembly was assigned to putative chromosomes, whereas placement of *D. subpalustris* contigs assigned 92.3% of the assembly (Fig. 2b). Our results reflect that *D. subpalustris* was more highly contiguous, with 17 contigs placed on Muller elements, including 7 contigs placed on chromosome X, 3 contigs placed on chromosome 2L, 2 contigs each placed on chromosomes 2R, 3L, and 3R, and 1 contig placed on chromosome 4. In contrast, the *D. palustris* assembly is more highly fragmented, with 218 contigs placed on Muller elements. However, the total assembly assigned was quite similar between *D. palustris* (154 Mbp), *D. subpalustris* (159 Mbp), and the reference *D. innubila* (158 Mbp). In fact, the more highly fragmented genome assemblies *D. recens* and *D. subquinaria* resulted in more unplaced contigs, highlighting the limits of the analysis. Altogether, these assignments provide a framework for chromosome-level comparisons within the quinaria species group and will enable downstream analyses of genome structure and evolutionary change across Muller elements.

To demonstrate the utility of assigning contigs to putative chromosomes, we compared synteny of our assigned contigs for quinaria group assemblies to the *D. innubila* chromosomal assembly using Chromeister (Pérez-Wohlfeil et al. 2019) (Fig. 2c). We used the *D. melanogaster* genome assembly as the outgroup for the analysis. *D. melanogaster* shared 81 syntenic blocks with *D. innubila,* with a median size of 748 kb. In contrast, *D. palustris* and *D. subpalustris* shared 183 and 165 syntenic blocks, respectively. Median syntenic block sizes were 914 kb for *D. palustris* and 998 kb for *D. subpalustris.* Notably, the median synteny block size was significantly greater in *D. subpalustris* compared to *D. palustris,* which we attribute to the higher assembly contiguity relative to *D. palustris.* The patterns observed in our genome-wide synteny comparison of the quinaria group genomes indicate that synteny block metrics are strongly influenced by assembly contiguity. Overall, our analysis revealed that the quinaria group genomes share synteny at the megabase scale, demonstrating the extent of conserved chromosomal architecture across the clade.

To visualize the syntenic relationships inferred by our contig assignments, we generated a GENESPACE riparian plot using shared single-copy BUSCO orthologs among *D. palustris, D. subpalustris, D innubila,* and the *D. melanogaster* outgroup (Fig. 2d). The plot demonstrates that the vast majority of assigned genomic regions map contiguously to their ancestral chromosome arms, with only one ambiguous ribbon connecting a *D. palustris* Muller element A contig to the *D. subpalustris* Muller element E. However, because the *D. palustris* and *D. subpalustris* contigs were arranged sequentially by size rather than anchored with physical scaffolding evidence, the internal shuffling observed within each Muller element likely represents technical artifacts rather than biological inversions. Nevertheless, our global synteny analysis demonstrates 2 major trends: (i) the Muller element structural features are conserved between the chromosome-level *D. innubila* and *D. melanogaster* genomes, suggesting that the quinaria clade likely does not feature major inter-Muller element rearrangements, and (ii) the quinaria clade in general features an expansion in the physical size of the X chromosome (Muller element A). Altogether, these visual patterns provide a strong validation for our chromosome-level contig assignments while continuing to highlight how genes rarely transfer between Muller elements within *Drosophila.*

### Updated phylogenomics and divergence time estimation of the quinaria species group

We completed our analysis of the quinaria group by investigating the evolutionary relationships between each species. We used BUSCO single-copy orthologs to construct a phylogenomic tree and estimate divergence times (Fig. 3, Table 5). We observed that the deep divergence between *D. melanogaster* and the quinaria group was estimated at 42.56 million years ago (MYA, 95% Highest Posterior Density (HPD) interval: 36.99 to 51.66 MYA), matching the established TimeTree consensus baseline (Kumar et al. 2022). Within the quinaria group, we estimated that the 2 major branches diverged approximately 17.86 MYA (95% HPD: 16.14 to 19.14 MYA), which is within the upper bound of the 10.40 to 18.39 MYA window previously reported (Spicer and Jaenike 1996; Morales-Hojas and Vieira 2012; Russo et al. 2013). At a finer taxonomic scale, our phylogenomic analysis estimated that *D. palustris* and *D. subpalustris* diverged approximately 2.19 MYA (95% HPD: 1.90 to 2.46 MYA), which is older than the 0.344 to 0.900 MYA range previously estimated (Spicer and Jaenike 1996; Russo et al. 2013). Because the above estimates were derived from amino acid sequences, we performed a similar analysis using a codon-based and third-codon position scheme, which may be more sensitive at more recent evolutionary divergence times (Breinholt and Kawahara 2013). Under the codon-based scheme, the divergence of *D. palustris* and *D. subpalustris* was estimated at 1.24 MYA (95% HPD: 0.93 to 1.57 MYA), while the third-codon position scheme estimated it at 1.23 MYA (95% HPD: 0.96 to 1.52 MYA). Additional estimates of divergence times for splits within the quinaria group and their comparison to previous estimates are included in Table 5. The complete, fully labeled phylogenetic topologies for all 4 molecular clock schemes are provided in Supplementary File 1.

**Fig. 3.**
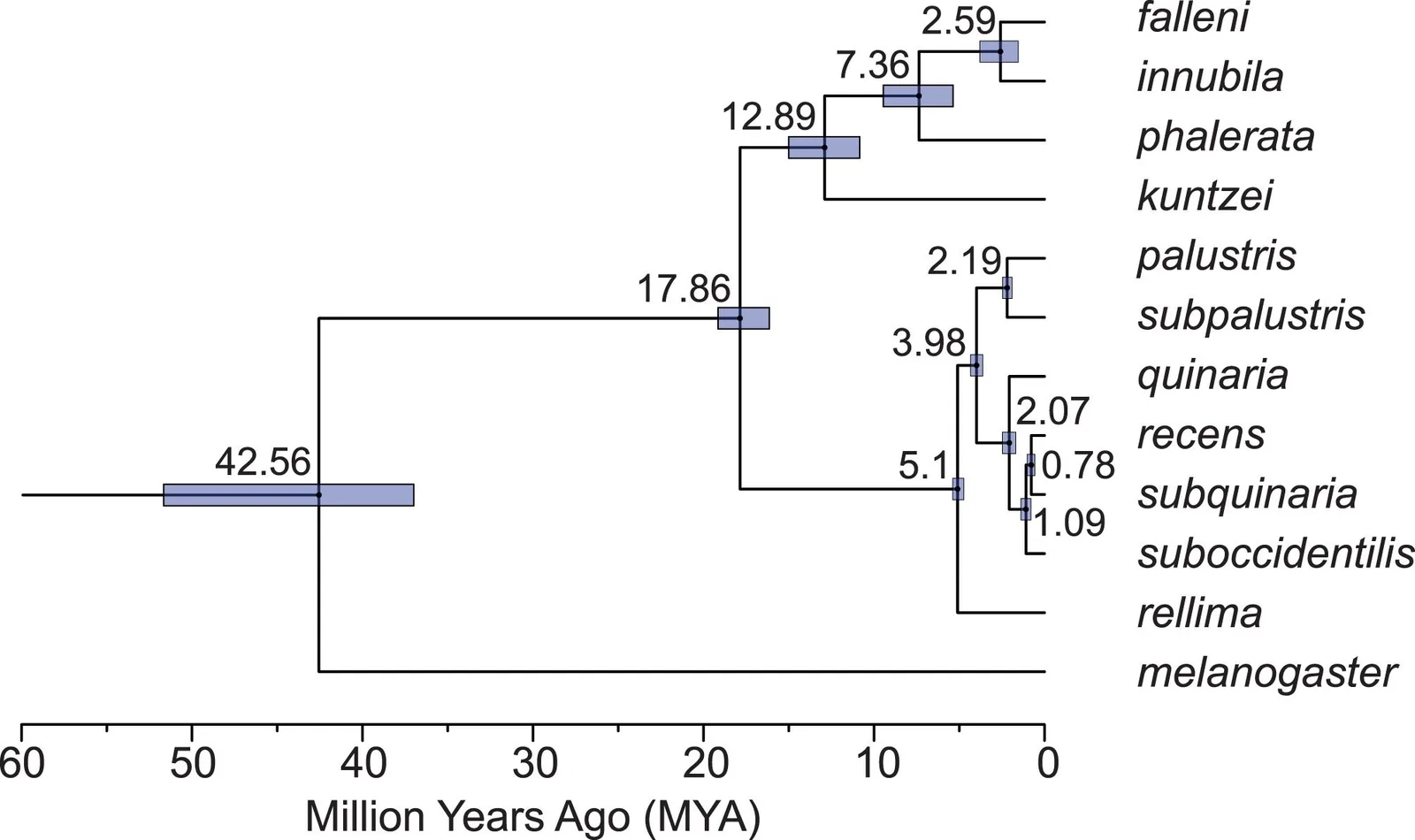
Time-calibrated phylogenomic tree of quinaria group species inferred using MCMCtree based on shared BUSCO single-copy orthologs. The scale bar at the bottom indicates evolutionary time in million years ago (MYA), and nodes are labeled with the mean divergence time. Horizontal bars represent the 95% highest posterior density (HPD) intervals for node ages. *D. melanogaster* served as the outgroup.

**Table 5.** Calculated phylogenomic distance vs TimeTree estimation range.

Node	TimeTree range (MYA)	Amino acid (95% HPD, MYA)	Codon (95% HPD, MYA)	3rd Position (95% HPD, MYA)	MCMCtree prior only (95% HPD, MYA)
1*—D. melanogaster* vs quinaria group	39.0 to 62.9*	42.56 (36.99 to 51.66)	41.43 (36.47 to 49.71)	43.63 (37.24 to 54.41)	42.75 (36.65 to 53.03)
2—(*kuntzei, phalerata, falleni, innubila)* vs *(rellima, palustris, subpalustris, quinaria, suboccidentalis, recens, subquinaria)*	10.40 to 18.39*	17.86 (16.14 to 19.14)	17.88 (15.97 to 19.14)	11.00 (9.06 to 13.69)	15.20 (11.25 to 18.75)
3—*D. kuntzei* vs (*phalerata, falleni, innubila)*	9.38 to 13.67	12.89 (10.84 to 15.00)	12.63 (10.36 to 14.86)	7.63 (5.73 to 9.57)	12.17 (6.53 to 18.06)
4—*D. rellima* vs (*palustris, subpalustris, quinaria, suboccidentalis, recens, subquinaria)*	-	5.10 (4.73 to 5.38)	5.08 (4.66 to 5.39)	5.19 (4.91 to 5.42)	4.68 (3.77 to 5.30)
5—(*palustris, subpalustris)* vs (*quinaria, suboccidentalis*, *recens, subquinaria)*	3.60 to 5.22*	3.98 (3.61 to 4.34)	3.23 (2.79 to 3.64)	3.23 (2.82 to 3.55)	3.51 (2.68 to 5.19)
6—*D. phalerata* vs (*falleni, innubila)*	5.90 to 9.51	7.36 (5.36 to 9.46)	8.63 (6.56 to 10.92)	4.12 (2.91 to 5.46)	8.67 (2.79 to 14.72)
7—*D. quinaria* vs (*suboccidentalis, recens, subquinaria)*	1.50 to 4.53	2.07 (1.68 to 2.47)	2.74 (2.36 to 3.16)	2.76 (2.41 to 3.09)	3.25 (1.84 to 4.74)
8—*D. falleni* vs *D. innubila*	4.28 to 4.93**	2.59 (1.54 to 3.79)	6.25 (4.29 to 8.06)	2.82 (1.96 to 3.84)	5.16 (1.55 to 10.35)
9—*D. suboccidentalis* vs (*recens, subquinaria)*	-	1.09 (0.80 to 1.38)	2.35 (2.00 to 2.75)	2.39 (2.07 -2.71)	2.53 (1.22 to 3.98)
*10—D. recens* vs *D. subquinaria*	2.56 to 2.95**	0.78 (0.56 to 1.01)	1.25 (1.00 to 1.53)	1.26 (1.05 to 1.49)	1.81 (0.86 to 3.11)
11—*D. palustris* vs *D. subpalustris*	0.344 to 0.900**	2.19 (1.90 to 2.46)	1.24 (0.93 to 1.57)	1.23 (0.96 to 1.52)	1.21 (0.18 to 2.26)

Divergence nodes (1 to 11) report estimated absolute divergence dates expressed in millions of years ago (MYA). The TimeTree Range column displays previously estimated divergence time ranges (Kumar et al. 2022). Subsequent columns represent new divergence time estimates generated in this study evaluating 4 distinct molecular evolutionary frameworks using MCMCtree: amino acid sequence, full codon positions, third-position degenerate codon sites, and an MCMCtree prior-only simulation framework to test data-independent probability boundaries. Asterisks (*) identify empirical TimeTree ranges utilized as rigid calibration anchors within the analytical model, while double asterisks (**) mark relaxed nodes calibrated using a wide 0.5× lower bound and 2.5× upper bound threshold.

Abbreviations: HPD, Highest Posterior Density interval spanning 95% of the statistical distribution boundary; MYA, millions of years ago.

The discrepancies between previous estimated divergence times and our estimates reveal distinct trends across the quinaria group. Within the deeper nodes on our phylogenomic tree, our estimates consistently shift toward older divergence times compared to previous estimates. As noted above, we date the split of the 2 major quinaria lineages (Node 2) at the uppermost bound of the TimeTree estimate. Conversely, at younger nodes our estimates do not match previous estimates. We observed that our divergence times of ∼2 to 3 MYA for the *D. falleni* and *D. innubila* split (Node 8) and ∼0.7 to 1.3 MYA for the *D. recens* and *D. subquinaria* split (Node 10) trended substantially more recent compared to the respective ∼4 to 5 MYA and ∼2 to 3 MYA estimates reported by TimeTree. In contrast, our *D. palustris* and *D. subpalustris* (Node 11) estimate placed the species divergence at ∼1 to 2 MYA, depending on the methodological scheme. The discrepancies likely reflect methodological differences in locus sampling density and sequence saturation. Our analysis leveraged 1,970 single-copy orthologs shared across all analyzed species, as compared to 1 to 6 mitochondrial or nuclear markers used in previous analyses (Spicer and Jaenike 1996; Morales-Hojas and Vieira 2012; Russo et al. 2013). Overall, our BUSCO-based phylogenomics analysis provides a more robust, updated evolutionary framework for the quinaria group.

## Conclusion

We generated high-quality genome assemblies for *D. palustris* and *D. subpalustris.* Repeat analyses revealed that both species contain relatively low repeat content compared to other *Drosophila* but consistent with other quinaria group genomes. We produced a draft gene annotation using RNA-seq evidence that identified 17,716 and 20,687 protein-coding genes, providing additional quantitative benchmarks for gene architecture in the clade. Synteny analysis based on BUSCO-guided contig assignment demonstrated extensive shared chromosomal structure across the quinaria group. Finally, BUSCO-based phylogenomics supported the evolutionary relationships within the group, confirming major branching points and refining divergence time estimates. Together, these results contribute to the expanding genomic framework available for Drosophilidae, facilitating future studies of genome evolution and the genetic basis for ecological and morphological diversity.

## Supplementary Material

jkag226_Supplementary_Data

## Data Availability

All data were deposited at NCBI under BioProject accession PRJNA1208480 for *D. palustris* and BioProject accession PRJNA1208482 for *D. subpalustris.* We used the following publicly available datasets for genomic comparisons: *D. melanogaster* (GCA_000001215.4), *D. falleni* (GCA_035045575.1), *D. innubila* (GCA_004354385.2), *D. kuntzei* (GCA_035042165.1), *D. phalerata* (GCA_951394115.2), *D. quinaria* (GCA_035047525.1), *D. recens* (GCA_037043495.1), *D. rellima* (GCA_035043635.1), *D. suboccidentalis* (GCA_037043435.1), and *D. subquinaria* (GCA_037043405.1). Supplemental material available at G3 online.
