# Supplementary material for: Draft hybrid assembly and annotation of the Drosophila palustris and Drosophila subpalustris genomes
Source: G3 (Bethesda). 2026 Aug 19;16(10):jkag226. doi: 10.1093/g3journal/jkag226 (PMC13643797; doi:10.1093/g3journal/jkag226)
Supplement: Supplementary file 1 [file jkag226_supplementary_data.zip › Supplementary_File_1_G3-2026-406991.pdf]

## Supplementary File 1

# Genome report: Draft hybrid assembly and annotation of the *Drosophila palustris and Drosophila subpalustris* genomes

William Dion, Emily Washeleski, Tessa E. Steenwinkel, Emily Taylor, Prajakta P. Kokate, Aidan VanDrie, Thomas Werner, Paul D. Goetsch*

## Introduction

This file contains the complete, fully labeled phylogenomic topologies and divergence time estimates for the quinaria group generated using MCMCtree. Four distinct molecular clock schemes are provided in standard NEXUS format: (1) Amino Acid sequence matrix, (2) Codon-based matrix, (3) Third-codon position matrix, and (4) the Prior-only run.

All branch lengths are scaled to absolute time in units of 100 million years. Nodes are annotated with metadata strings containing the 95% Highest Posterior Density (95% HPD) interval boundaries, representing the upper and lower limits of the Bayesian divergence estimation. These strings are fully compatible with phylogenetic visualization software such as FigTree.

## Tree 1: Amino Acid Dataset

BEGIN TREES;

UTREE 1 = (((((Dfal: 0.025856, Dinn: 0.025856) [&95%HPD={0.0154329, 0.0379311}]: 0.047755, Dpha: 0.073610) [&95%HPD={0.0536319, 0.0945629}]: 0.055309, Dkun: 0.128919) [&95%HPD={0.108393, 0.150006}]: 0.049664, (((Dpal: 0.021886, Dspa: 0.021886) [&95%HPD={0.0189954, 0.0245569}]: 0.017956, (Dqui: 0.020664, ((Drec: 0.007771, Dsqu: 0.007771) [&95%HPD={0.0056342, 0.010126}]: 0.003120, Dsoc: 0.010892) [&95%HPD={0.0079861, 0.0138257}]: 0.009772) [&95%HPD={0.0168052, 0.0246538}]: 0.019178) [&95%HPD={0.0361267, 0.0434453}]: 0.011159, Drel: 0.051001) [&95%HPD={0.0472756, 0.0538094}]: 0.127583) [&95%HPD={0.161426, 0.191442}]: 0.246982, Dmel: 0.425566) [&95%HPD={0.369937, 0.516622}];

END;

## Tree 2: Codon Dataset

BEGIN TREES;

UTREE 1 = (((((Dfal: 0.062463, Dinn: 0.062463) [&95%HPD={0.0428689, 0.0806085}]: 0.023865, Dpha: 0.086328) [&95%HPD={0.0656155, 0.109153}]: 0.039941, Dkun: 0.126269) [&95%HPD={0.103568, 0.148586}]: 0.052501, (((Dpal: 0.012443, Dspa: 0.012443) [&95%HPD={0.0093323, 0.0156875}]: 0.019438, (Dqui: 0.027400, ((Drec: 0.012487, Dsqu: 0.012487) [&95%HPD={0.0099519, 0.0152555}]: 0.011026, Dsoc: 0.023513) [&95%HPD={0.020041, 0.0274556}]: 0.003887) [&95%HPD={0.023644, 0.0316054}]: 0.004481) [&95%HPD={0.0278524, 0.036397}]: 0.018880, Drel: 0.050760) [&95%HPD={0.0466157, 0.0539349}]: 0.128010) [&95%HPD={0.159707, 0.191374}]: 0.235499, Dmel: 0.414269) [&95%HPD={0.364706, 0.49712}];

END;

## Tree 3: Third-Codon Position Dataset

BEGIN TREES;

UTREE 1 = (((((Dfal: 0.028218, Dinn: 0.028218) [&95%HPD={0.0195942, 0.0384338}]: 0.012963, Dpha: 0.041181) [&95%HPD={0.0291227, 0.0546439}]: 0.035075, Dkun: 0.076256) [&95%HPD={0.057272, 0.0957068}]: 0.033721, (((Dpal: 0.012259, Dspa: 0.012259)

[&95%HPD={0.0095924, 0.0151659}]: 0.019803, (Dqui: 0.027577, ((Drec: 0.012614, Dsqu: 0.012614) [&95%HPD={0.010454, 0.0148768}]: 0.011311, Dsoc: 0.023925) [&95%HPD={0.0207052, 0.0270875}]: 0.003652) [&95%HPD={0.024077, 0.0309499}]: 0.004484) [&95%HPD={0.0282398, 0.0355464}]: 0.019823, Drel: 0.051884) [&95%HPD={0.049141, 0.0542103}]: 0.058093) [&95%HPD={0.0905932, 0.136914}]: 0.326287, Dmel: 0.436264) [&95%HPD={0.372438, 0.544111}];

END;

## Tree 4: Prior Only

BEGIN TREES;

UTREE 1 = (((((Dfal: 0.051628, Dinn: 0.051628) [&95%HPD={0.0155389, 0.103477}]: 0.035030, Dpha: 0.086658) [&95%HPD={0.0279326, 0.14721}]: 0.035060, Dkun: 0.121718) [&95%HPD={0.0652573, 0.180636}]: 0.035010, (((Dpal: 0.012090, Dspa: 0.012090) [&95%HPD={0.0018109, 0.0225817}]: 0.027564, (Dqui: 0.032465, ((Drec: 0.018108, Dsqu: 0.018108) [&95%HPD={0.0085878, 0.0310825}]: 0.007168, Dsoc: 0.025276) [&95%HPD={0.0121858, 0.0397838}]: 0.007189) [&95%HPD={0.0184404, 0.0474023}]: 0.007189) [&95%HPD={0.0267831, 0.0519118}]: 0.007150, Drel: 0.046804) [&95%HPD={0.0377357, 0.0529895}]: 0.109924) [&95%HPD={0.112517, 0.187546}]: 0.270757, Dmel: 0.427485) [&95%HPD={0.366497, 0.530278}];

END;
